# Proteomics and functional study reveal kallikrein-6 enhances communicating hydrocephalus

**DOI:** 10.1186/s12014-021-09335-9

**Published:** 2021-12-16

**Authors:** Lei Yuan, Dongdong Zou, Xia Yang, Xin Chen, Youming Lu, Aijun Zhang, Pengqi Zhang, Fance Wei

**Affiliations:** Department of Neurosurgery, The Affiliated Sixth People’s Hospital, Shanghai Jiaotong University, NO. 600 Yishan Road, Shanghai, 200233 China

**Keywords:** Cerebrospinal fluid, Communicating hydrocephalus, Kallikrein-6, Quantitative proteomic

## Abstract

**Background:**

Communicating hydrocephalus (CH) is a common neurological disorder caused by a blockage of cerebrospinal fluid. In this study, we aimed to explore the potential molecular mechanism underlying CH development.

**Methods:**

Quantitative proteomic analysis was performed to screen the differentially expressed proteins (DEPs) between patients with and without CH. A CH rat model was verified by Hoechst staining, and the co-localization of the target protein and neuron was detected using immunofluorescence staining. Loss-of-function experiments were performed to examine the effect of KLK6 on the synapse structure.

**Results:**

A total of 11 DEPs were identified, and kallikrein 6 (KLK6) expression was found to be significantly upregulated in patients with CH compared with that in patients without CH. The CH rat model was successfully constructed, and KLK6 was found to be co-localized with neuronal nuclei in brain tissue. The expression level of IL-1β, TNF-α, and KLK6 in the CH group was higher than that in the control group. After knockdown of KLK6 expression using small-interfering RNA (siRNA), the expression levels of synapsin-1 and PSD95 in neuronal cells were increased, and the length, number, and structure of synapses were significantly improved. Following siRNA interference KLK6 expression, 5681 differentially expressed genes (DEGs) were identified in transcriptome profile. The upregulated DEGs of Appl2, Nav2, and Nrn1 may be involved in the recovery of synaptic structures after the interference of KLK6 expression.

**Conclusions:**

Collectively, KLK6 participates in the development of CH and might provide a new target for CH treatment.

**Supplementary Information:**

The online version contains supplementary material available at 10.1186/s12014-021-09335-9.

## Introduction

Communicating hydrocephalus (CH), a common neurological disorder, is associated with cognitive decline and severe deterioration of the quality of life of patients. CH is known to be etiologically heterogeneous and can be caused by subarachnoid hemorrhage, leptomeningeal metastasis, trauma, and tumors [1]. The probability of CH after subarachnoid hemorrhage is as high as 67% and as low as 13% [2]. Although the brain is less prone to hydrocephalus because of the compensatory absorption of cerebrospinal fluid (CSF), it is irreversible once it occurs, and patients often require long-term shunt therapy [3]. Carelessness related to CH can lead to death or disability. Subarachnoid space fibrosis leads to the development of multiple forms of CH due to impaired CSF flow and reduced CSF drainage [4]. However, more than 100 years after the first experimental study of hydrocephalus, the molecular mechanism of CH following subarachnoid hemorrhage (SAH) remains unknown.

The human kallikrein 6 (*KLK6*) gene encodes a member of the KLK-related peptidase family, also known as the human cancer biomarker family, which comprises at least 15 members and is located on chromosome 19q13.3–13.4 [5]. Currently, quantitative proteomics is usually employed to identify differentially expressed proteins (DEPs) in specific biological backgrounds. Its combination with mechanistic experiments allows effective exploration of the potential functions of KLK proteins. Kryza et al. identified the involvement of KLK14 in the aggressive characteristics of prostate cancer using a method called cell-secreted proteomics [6]. In-depth proteomic and biochemical studies on cervical–vaginal fluid demonstrated that KLK was involved in the regulation of the female reproductive system [7]. However, there have been no studies on the application of proteomics to study KLK6-related functions in CH. The expression of KLK6 in central nervous system (CNS) diseases is heterogeneous, such as upregulation of KLK6 expression in CNS inflammatory response and downregulation of KLK6 expression in Alzheimer’s disease (AD) and Parkinson’s disease [8]. A previous study showed that KLK6 is expressed in aneurysmal SAH and as a serum prognostic biomarker [9].

*KLK6* encodes an enzyme with trypsin-like properties that degrades the extracellular matrix [10]. The abnormal decline in extracellular matrix protein in CSF is associated with various neurological diseases, such as SAH [11], stroke [12], and traumatic brain injury [13]. These findings suggest that KLK6 plays a vital role in neurological diseases, but its detailed function in CH after SAH remains unclear. Therefore, we aimed to explore the effects of KLK6 on neurons by quantitative proteomics and functional loss analysis and provide a theoretical basis and therapeutic target for CH prevention and treatment.

## Material and methods

### Ethics statement

The human and rat study protocol was adhered to the principles of the Declaration of Helsinki and approved by the Institutional Ethics Committee of The Affiliated Sixth People’s Hospital, Shanghai Jiaotong University. All participants were fully informed and signed written informed consent in this study. All efforts were made to minimize rat suffering.

### Patients

The data of patients with cerebral hemorrhage accompanied by CH (n = 12) and of patients with cerebral hemorrhage without CH (n = 8) were collected. The clinical profiles of patients with SAH are shown in Additional file 3: Table S1, and no statistical differences were observed between the CH and control groups regarding age, sex, and comorbidities of patients in our study (Additional file 4: Table S2). The patients were diagnosed according to the standard clinical and laboratory criteria and referred from our neurology department. The following cases were excluded: normal-pressure hydrocephalus due to normal production; hydrocephalus due to overproduction, such as choroid plexus papilloma evacuation of hydrocephalus; or cases of reduced volume secondary to brain parenchyma. Of these, three patients and five healthy controls were included in quantitative proteomics. The remaining 9 samples of patients with CH and 3 control samples were used for ELISA.

### Protein extraction

Proteins were extracted from CSF samples of three patients with CH and five patients without CH as previously described [14]. Briefly, the BCA kit (Thermo Scientific, USA) was used to extract and measure the protein concentration. About 50 μg protein from each sample was reduced with 10 mM dithiothreitol for 2–3 h at 37 °C. Then sample was loaded on 10 KD centrifugal filter tubes (Millipore) and centrifuged for 40 min at 12,000*g*. Samples were further alkylated with 30 mM iodoacetamide in the darkness for 30 min and centrifuged at 12,000*g* for 40 min. Then wash the sample with 100 mM ammonium bicarbonate two times. Proteins were digested at 37 °C for 18 h with trypsin (Promega) at a concentration of 1:50 (w:w, trypsin to protein) in 100 mM ammonium bicarbonate. The tryptic peptides were collected by centrifugation at 12,000*g* and desalted via StageTipC18 (3 M Empore, USA). Finally, the proteins were separated into fractions using SDS-PAGE and unqualified samples (The protein patterns and concentrations were significantly different from those of other groups) by SDS-PAGE were removed in this step.

### Nano liquid chromatography tandem mass spectrometry (LC–MS/MS) analysis

All experiments of LC–MS/MS analysis were performed on a Q-Exactive mass spectrometer with an ancillary EASY-nLC 1000 HPLC system (Thermo Fisher Scientific). Q-Exactive was operated under positive ionization mode (ESI, The Nano Flex Ion Source) and data-dependent acquisition mode. The tryptic digested peptides were loaded on a 75 μm × 200 mm fused silica column packed in-house with 3 μm ReproSil-Pur C18 beads (Dr. Maisch GmbH, Ammerbuch, Germany) and separated with a 120-min gradient at a flow rate of 300 nL/min. Solvent A contained 100% H_2_O and 0.1% formic acid; Solvent B contained 100% acetonitrile and 0.1% formic acid. The gradient was 2–4% B, 1 min; 4–27% B, 95 min; 27–35% B, 15 min; 35–90% B, 1 min, 90% B, 8 min. The MS instrument parameters were: MS1 full scan resolution, 70,000 at m/z 200; automatic gain control target, 1 × 106; maximum injection time, 50 ms. MS2 scan resolution 17,500 at m/z 200; automatic gain control target, 1 × 105; maximum injection time, 100 ms; isolation window, 2.0 m/z; dynamic exclusion, 30 s. The precursor ions were fragmented by higher energy collisional dissociation (HCD) with a normalized collision energy of 27%.

### Protein identification and analysis

The raw MS2 data were searched using Maxquant version 1.6.0.1 (Thermo Scientific) against the human UniProt database. Carbamidomethyl (Cys) was set as a fixed modification. The variable modifications included oxidation (Met) and acetyl (protein N-term). A positive identification of peptide length was required to contain a minimum of seven amino acids and a maximum of one peptide PEP. The tolerances of MS/MS were set as 20 ppm. The false discovery rates (FDRs) of the peptide-spectral match and protein identification were set as 0.01. The protein intensity is derived from label-free quantification intensity generated by MaxQuant. Proteins with a P-value of < 0.05 were considered DEPs. Finally, Gene Ontology (GO) functional and Kyoto Encyclopedia of Genes and Genomes (KEGG) pathway enrichment analysis were applied on DEPs, and DEPs were corrected using principal component analysis (PCA) via the pipeline process. The spectral data were deposited to the Figshare website (https://figshare.com/account/login) with the dataset https://doi.org/10.6084/m9.figshare.12335288.

### Enzyme-linked immunosorbent assay (ELISA) analysis

The expression levels of KLK6 in CSF from 9 patients with CH and 3 patients without CH were measured using a commercial ELISA 96 T Kit (Boster Biological Technology, China) according to the manufacturer’s instructions. While only 6 blood samples were collected from above 9 CH patients and also used for ELISA analysis, and this experiment was conducted in duplicate. In addition, the concentration of Tumor necrosis factor alpha (TNF-α, YIFEIXUE BIOTECH, Nanjing, China NO.YFXER00038) and interleukin-1β (IL-1β, YIFEIXUE BIOTECH, Nanjing, China NO.YFXEM00028) in the brain parenchyma and CSF of rats were detected by ELISA kit, according to the instructions of the manufacturer. The absorption value was measured at a wavelength of 450 nm using a microplate reader, and the expression level was calculated using standards.

### Rat model and sample collection

Wistar rats were purchased from Yingbio (Shanghai, China), and thirty 12-week-old male Wistar rats were randomly divided into a healthy control group and a model group. A CH rat model was constructed according to a previously described method [15, 16]. Briefly, citrated rat venous blood and artificial CSF (ACSF) were injected stereotactically into the lateral cerebral ventricles of the model group and the lateral ventricle of the control group, respectively. The rats in the two groups were fed for 3 weeks in the same environment and weighed daily. After 3 weeks, they were sacrificed to obtain CSF and brain tissue, which was used for ELISA analysis and paraffin-embedded for subsequent staining.

### Hoechst staining

Hoechst staining was used to observe the apoptosis and morphological changes of neurons. Paraffin-embedded brain tissues were cut into 4-μm-thick slices using a rotary microtome (RWD Life Science co., ltd) and stained using the Hoechst staining kit (Beyotime, Shanghai). The slices were rinsed twice with phosphate buffer solution for 3 min each time. After adding Hoechst staining solution, the paraffin slices were incubated at 37 °C for 10 min and detected under a fluorescence microscope. The nuclei of apoptotic cells showed strong fluorescence, whereas the nuclei of nonapoptotic cells showed weak fluorescence.

### Cell culture and knockdown of KLK6 expression using small interfering RNA (siRNA)

Primary rat hippocampal neuron cells were purchased from Procell Life Science & Technology (Wuhan, China) and cultured in a Cell Culture Flask (#353108, FALCONNest) containing RPMI medium (#CM-R107, Procell) at 37 °C with 5% CO_2_. Three siRNA targeting KLK6 and one non-targeting control were synthesized by Gibico (Shanghai, China), as shown in Additional file 5: Table S3. According to the manufacturer’s instructions, the KLK6 siRNA or the control siRNA were transfected into neurons for 48 h using Lipofectamine 2000 (Invitrogen). The transfection efficiency was determined using qRT-PCR and western blotting.

### Immunofluorescence staining

The transfected neurons grew adherent to the slides and were fixed with 4% paraformaldehyde for 10 min. The slides and 4-mm-thick paraffin-embedded sections were used for immunofluorescence staining. Briefly, the slides and paraffin sections were permeated with 0.1% Triton X-100 and sealed with 3% bovine serum albumin, followed by incubation with primary antibodies overnight. After washing them three times, the sections were incubated with secondary antibodies for 2 h and then stained with DAPI (Invitrogen, Carlsbad, CA, USA) for 15 min at room temperature. Finally, the sections were imaged under a microscope. Rat tissue sections were prepared with KLK6 (1:250; Santa Cruz, China) and neuronal nuclei (NeuN) (1:300; Cell Signaling, USA) proteins as primary antibodies, and the neuron lines were treated with microtubule-associated protein 2 (MAP-2) (1:500; Signalway Antibody LLC, USA) protein as a primary antibody.

### Western blotting

The total proteins of cultured neurons were obtained using RIPA (Thermo, USA) to lysate and measured using the BSA kit (TaKaRa, Dalian). Next, aliquots containing 20 μg of protein were electrophoresed on 10% SDS-PAGE and transferred onto PVDF membranes (ThermoScientific, Madison, WI, USA). The PVDF membranes were blocked with TBST solution containing 5% skim milk at room temperature for 3 h and incubated with primary antibodies (including anti-KLK6, anti-PSD95, and anti-YN1-specific antibodies; the dilution ratio was 1:1000) overnight at 4 °C and with HRP-conjugated secondary antibody (1:10,000) at room temperature for 1 h. The protein bands were detected using a high-sensitivity ECL luminescence kit (ThermoScientific, Madison, WI, USA) and imaged using a chemiluminescence imaging analysis system.

### Quantitative reverse transcription PCR (qRT-PCR)

TRIzol reagent (Invitrogen Life Technologies, Inc.) was used to extract total RNA from neurons according to the manufacturer’s protocol. The RNA concentration and purity were determined using a microspectrophotometer (Tiangen Biotech Co., Ltd.). RNA was reversely transcribed into first-strand cDNA using the RevertAid First-Strand cDNA synthesis kit (ThermoScientific, Madison, WI, USA). According to the manufacturer’s instruction, the cDNA was amplified using FastStart Universal SYBR Green Master mix on a QuantStudio 6 Flex Real-Time PCR System (Thermo Fisher Scientific, Inc.). All primers used in this study were synthesized by Sangon Biotech (Shanghai, China) (Additional file 5: Table S3). GAPDH served as a housekeeping gene, and gene expressions were normalized using the 2^−ΔΔCq^ method.

### Transcriptome sequencing of neuron

Total RNA was isolated from primary rat hippocampal neuron cells transfected with siRNA-KLK6 (n = 3) or siRNA-NC (n = 3) using TRIzol reagent. The RNA concentration and purity were determined using a microspectrophotometer. Ribosomal RNA was eliminated from total RNA, and the remaining RNA was used to construct the cDNA library using the TruSeq Stranded RNA Sample Preparation Kit (Illumina, San Diego, CA, USA). Then, the cDNA library was purified on the AMPure XP system, and the quality of the library was assessed using an Agilent Bioanalyzer 2100 system. Finally, the cDNA library was subjected to RNA sequencing on an Illumina HiSeq 2500 platform with a paired-end 150-bp read run. FastQC (http://www.bioinformatics.babraham.ac.uk/projects/fastqc/) was used for the quality control of raw reads. The gene expression was normalized by fragments per kilobase of exon per million fragments mapped. The DEGSeq algorithm was utilized to screen differentially expressed genes (DEGs, with a significance threshold of Log2FC > 1 or <  − 1 and FDR < 0.05. The DEGs were utilized for functional annotation and enrichment analysis of GO and KEGG, using an R-based hypergeometric distribution. The RNA-seq data generated in this study is available in National Center for Biotechnology Information (NCBI) under accession number PRJNA719985.

### Statistical analysis

All data were analyzed using SPSS version 17.0 and presented as the mean ± SD. Student’s *t*-test was used for comparisons between the two groups. P < 0.05 indicated statistical significance.

## Results

### Overall analysis of quantitative proteomics

We initially included three patients with CH samples and five patients without CH samples (non-CH) for quantitative proteome analysis to avoid data loss of credibility due to insufficient sample repetition. The SDS-PAGE results showed (Additional file 1: Fig. S1) that protein patterns from two non-CH controls were unreliable, at which point only the remaining three groups were used for subsequent analysis. The peptide distribution, relative protein molecular weight, peptide length, and proteins sequence coverage are shown in Additional file 2: Fig. S2. These results provided the confidence of our MS2 data. We obtained 388,652 total spectra, and the number of spectra matched by the identified peptide segment was 41,139. Among them, a total of 6815 peptides and 1008 protein groups were identified (Additional file 6: Table S4).

### Occurrence of CH alters proteome in CSF

**Fig. 1:**
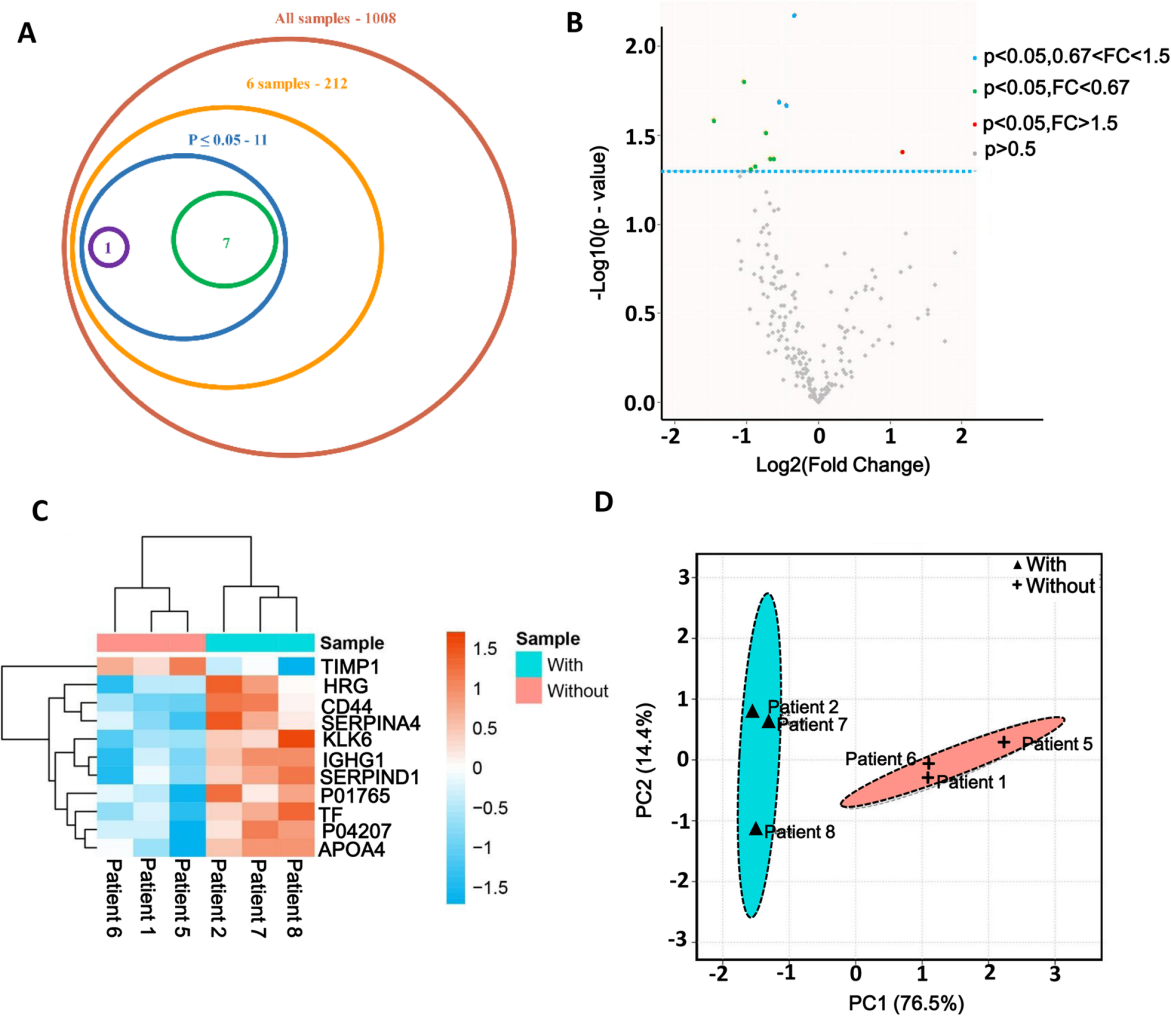
Identified DEPs between non-CH (n = 3) and CH (n = 3). **A** Number of proteins identified. A total of 1008 proteins were identified from six samples; After decontamination, 212 proteins had quantitative information in six samples. Only 11 proteins were defined as DEPs, reaching P < 0.05. Among them, 10 proteins were upregulated and one protein was downregulated in the CH group, compared with the non-CH group. **B** Volcano plot of DEPs. **C** Heatmap for significantly DEPs. The full name of the DEPs can be found in Additional file 7: Table S5. D: PCA plots of 11 DEPs between non-CH and CH. “With” indicates patients with CH and “without” indicates patients without CH

Notably, a total of 1008 protein groups were identified in six samples (Fig. 1A). For protein quantitation, reverse and contaminant proteins were removed, after that, we acquired 212 proteins with quantitative information in six samples (Fig. 1A). In addition, a total of 11 significant DEPs were identified, among them, the expressions of 10 proteins were upregulated and 1 was downregulated in the CH group compared with those in the non-CH group (Fig. 1B). The specific information of 11 DEPs is shown in Additional file 7: Table S5. The DEPs in the non-CH and the CH groups were clustered into two groups with different expression patterns (Fig. 1C). Further, given the differences in protein signature between different samples, we conducted a PCA analysis of the DEPs. The six components explained 100% of the dataset variation, as shown in Fig. 1D. The principal components PC1 and PC2 explained 76.5% and 14.4% of the total variation, respectively. There were considerable differences in proteins between the two groups, and the result indicated that the proteome was significantly altered after the occurrence of CH.

### KLK6 is highly expressed in patients with CH

**Fig. 2:**
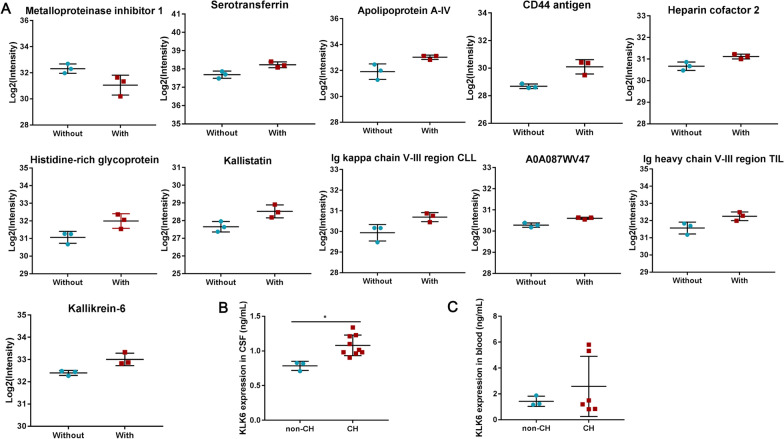
Intensity analysis of DEPs. **A** The intensity of DEPs was derived from the expression level of the non-CH group compared with the CH group. “With” represents the group of the CH, and “without” represents patients without CH. **B** KLK6 protein levels in cerebrospinal fluid from CH patients (n = 9) and non-CH patients (n = 3) was measured by ELISA. **C** KLK6 protein levels in blood from CH patients (n = 7) and non-CH patients (n = 3) was measured by ELISA. *means P < 0.05

The expression intensity of 11 DEPs in six samples was determined by the ratio of the expression level of the non-CH group to that of the CH group and represented as the value of fold-change (FC). The results showed that only metalloproteinase inhibitor 1 had an FC value of > 1.5 (Fig. 2A). This result indicates that metalloproteinase inhibitor 1 expression was downregulated in CH, whereas the expressions of the remaining 10 DEPs were upregulated. Those 10 DEPs included CD44 antigen, Ig heavy chain V-III region TIL/TUR/WAS/POM, histidine-rich glycoprotein, Ig kappa chain V-III region CLL, apolipoprotein A-IV, kallistatin, and KLK6 (Fig. 2A). These 11 DEPs were mainly involved in chemotaxis (GO: 0006935) and the HIF-1 signaling pathway (Additional file 8: Table S6). KLK6 is known to be specificity enhanced in the brain and abnormally expressed in many neurological diseases, and its role in CH is still unclear [17]. Therefore, we further investigated the function and mechanism of KLK6 in CH. We detected KLK6 expression in CSF from 9 cerebral hemorrhage patients with CH (CH group) and 3 patients without CH (non-CH group) to determine whether KLK6 was differentially expressed in patients using ELISA. As shown in Fig. 2B, compared with the non-CH controls, in patients with CH, the protein expression of KLK6 was significantly upregulated in CSF, whereas there was no significant difference in the blood (Fig. 2C). In summary, these results indicated that KLK expression was abnormally upregulated in patients with cerebral hemorrhage with CH, suggesting that KLK6 is involved in the progression of CH.

### KLK6 is highly expressed in neurons of CH model rat

**Fig. 3:**
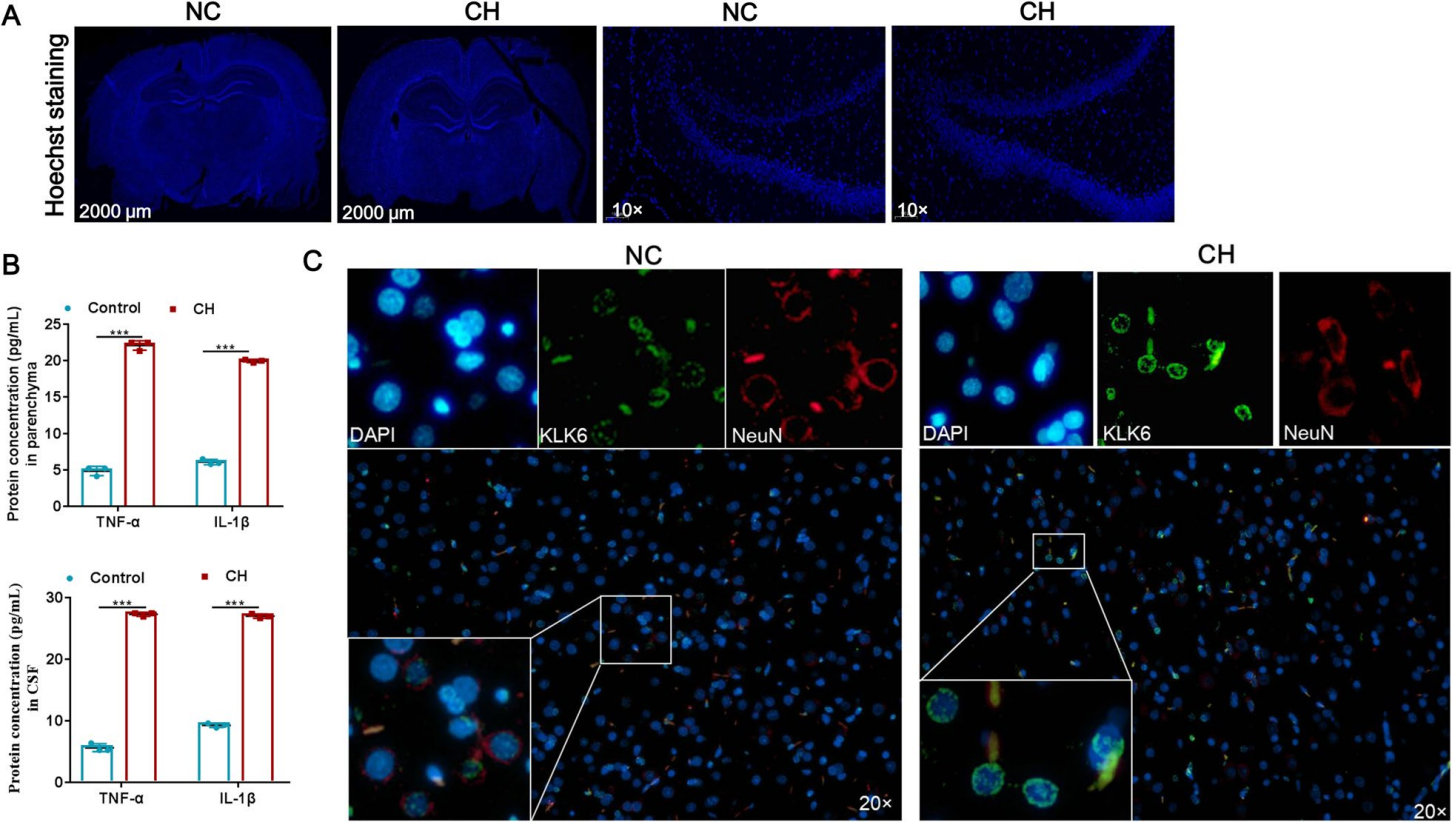
Expression of KLK6 and neurons in brain tissue. **A** Hoechst staining of brain tissue of rat (Left: scale bar was 2000 μm for whole brain; Right: magnification was 10× for partial brain tissue). B: The concentration of IL-1β and TNF-α in brain parenchyma (n = 3) and CSF (n = 3) were detected by using ELISA. ***indicates P < 0.001. **C** Double immunofluorescence staining of KLK6 in rat cortical neurons (magnification was 20×)

To further explore the function and mechanism of KLK6 in vivo, we constructed the CH model by injecting citrated rat venous blood or ACSF into the lateral cerebral ventricles. Using Hoechst staining, the anterior fontanelle and posterior 0.4-mm coronal lateral ventricle with widths > 1 mm were characterized as hydrocephalus. The brain tissue morphology of rats injected with venous blood reached the standard of hydrocephalus 3 weeks after surgery, which showed significant enlargement of the lateral ventricle, cortex thinning, mild hippocampus compression, and cell apoptosis (Fig. 3A). However, all rats injected with ACSF exhibited no ventricular enlargement, cortical compression, or thinning (Fig. 3A). Pro-inflammatory cytokine released in CSF after SAH is a typical pathological feature [18], thus we detected the expression of pro-inflammatory factors IL-1β and TNF-α in the brain parenchyma and CSF of rats. The results of ELISA showed that the concentration of IL-1β and TNF-α were increased in the CH group in both parenchyma and cerebrospinal fluid, compared with the control group (Fig. 3B). Previous research showed that the neurons and synapses of rats underwent significant pathological changes after CH occurred and that the abnormal function of neurons was closely related to CH [16]. Thus, KLK6 expression in neurons was identified. Immunofluorescence staining showed that KLK6 mainly located in nucleus, and co-localized with NeuN in brain tissue, and that the KLK6 expression in brains injected with venous blood was higher than that in brains injected with ACSF (Fig. 3C). These results indicated that the KLK6 was highly expressed in neurons of the CH model rats.

### KLK6 inhibits synaptic protein expression

**Fig. 4:**
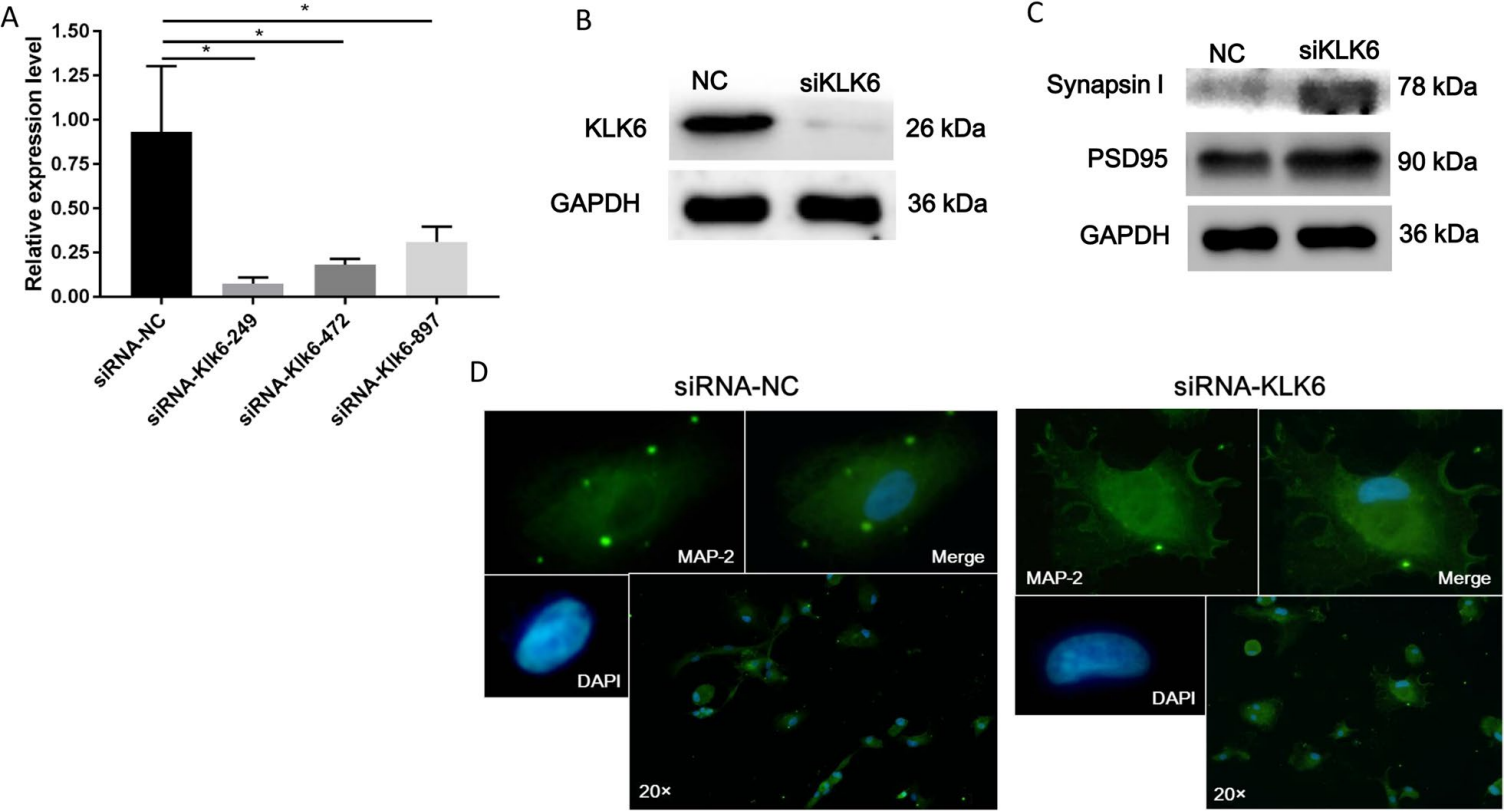
Effect of KLK6 on neuronal function. Efficiency of siRNA interference of KLKL6 expression in neurons was detected using qRT-PCR (**A**) and western blotting (**B**). **C** The expression levels of synapsin-1 and PSD95 were determined after interference of KLKL6 using western blotting. **D** Immunofluorescence staining of MAP-2 in rat cortical neurons (magnification was 20×)

The synapse is the basic unit that maintains neuronal signaling, and it is structurally stable during healthy adulthood [19]. Our previous study proved that the length and number of axons and dendrites of neurons in an animal model of CH were significantly reduced [16]. Therefore, we further investigated whether KLK6 induces changes in the expression of structural proteins in the synapses of neurons. For this purpose, we designed three siRNA sequences and one control sequence and efficiently detected the interference. The mRNA and protein expression levels of KLK6 in the neuronal cells significantly decreased after siRNA-249 treatment compared with those in the control group (Fig. 4A and B). Hence, siRNA-249 was used in subsequent experiments.

Synapsin-1 is a presynaptic protein, and PSD95 is a post-synaptic protein that marks the density of synaptic proteins [20]. Thus, both synapsin-1 and PSD95 were used to evaluate the structure of synapses in this study. As shown in Fig. 4C, the expression levels of PSD95 and synapsin-1 were significantly elevated in the KLK6-siRNA-249 group compared with that in the control group. These findings indicate that KLK6 inhibits synaptic protein expression. Moreover, MAP-2 is necessary to maintain microtubule stability and guide microtubule dynamics and is considered a marker of neuron and microtubulin interaction [21]. MAP-2-labeled immunofluorescence staining was performed to detect the number and length of synapses in neurons after the interference of KLK6 expression using siRNA. The fluorescence signal of MAP-2 significantly increased with KLK6-siRNA-249 treatment, along with the length and number of synapses (Fig. 4D). These results suggested that siRNA interference of KLK6 expression protected against injury to the synapse structure.

### siRNA-KLK6 alters the expression profile of neurons

**Fig. 5:**
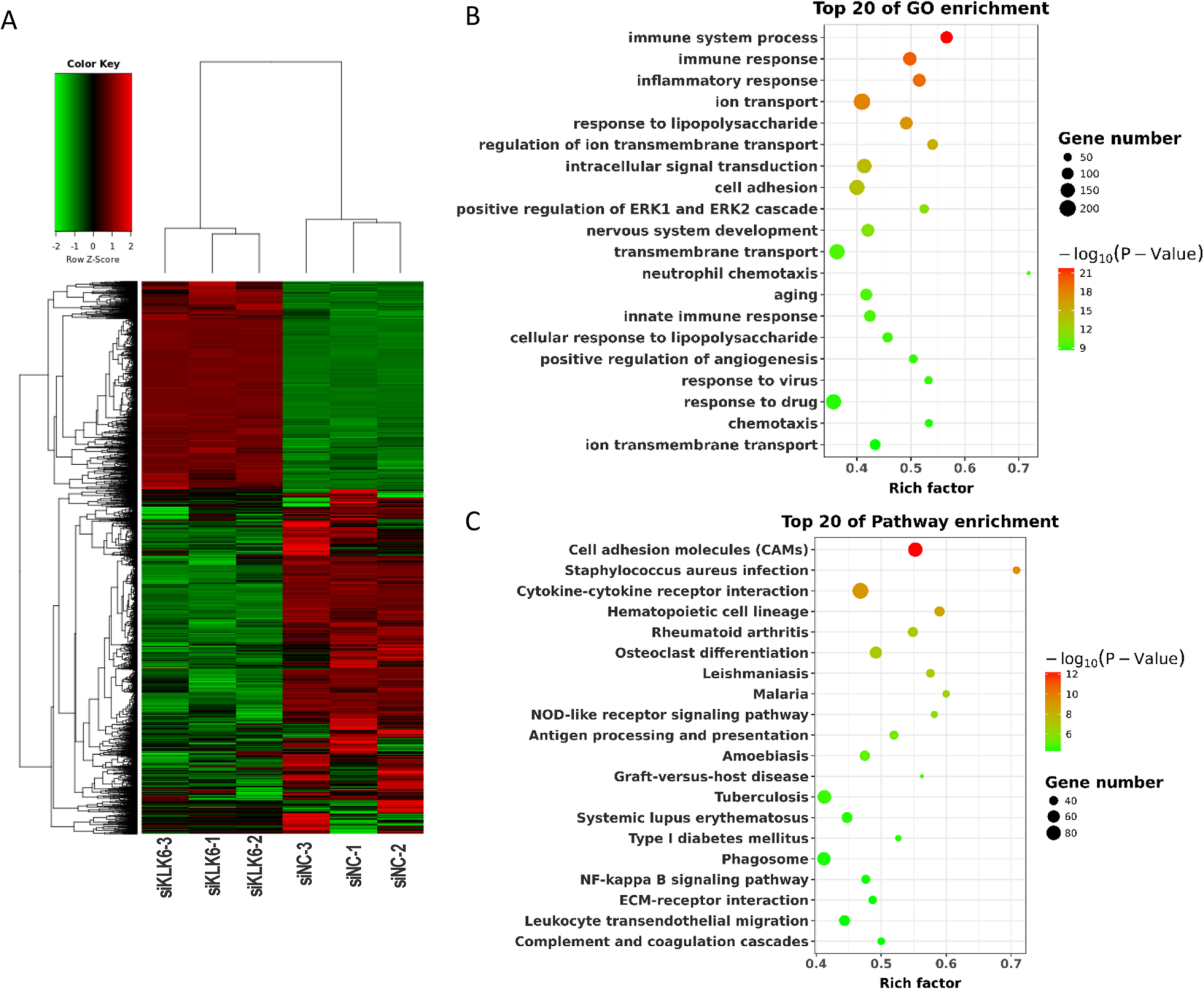
Transcriptome profile after interference of KLK6 expression. **A** Clustering heatmap for differentially expressed genes (DEGs) in neurons after transfection with siRNA-KLK6 (n = 3) or siRNA-NC (n = 3). Red and green represent upregulated and downregulated DEGs expressed in the siRNA-KLK6 group compared with the siRNA-NC group. **B** Top 20 GO enrichments for DEGs. **C** Top 20 pathway enrichments for DEGs

Transcriptome sequencing was performed on the neurons after transfection with siRNA-KLK6 (n = 3) or siRNA-NC (n = 3) to further investigate the mechanism underlying the KLK6-mediated inhibition on neuron cells. The quality control of transcriptome sequencing is shown in Additional file 9: Table S7. As a result, siRNA interference of KLK6 expression resulted in the abnormal expression of 5861 genes, among which, there were 2205 upregulated DEGs and 3658 downregulated DEGs in the siRNA-KLK6 group compared to the siRNA-NC group, and these DEGs were clustered into two branches (Fig. 5A). GO functional analysis showed that these DEGs were mainly enriched in GO terms associated with immune and cellular junction, for instance, immune system process, innate immune response, and cell adhesion (Fig. 5B). KEGG pathway analysis showed that the DEGs were mainly involved in cellular junction-related pathways, such as cell adhesion molecules and the ECM-receptor interaction pathway (Fig. 5C). These results indicate that KLK6 inhibition using siRNA contributes to protecting against damage to the synapse structure by neuronal immune regulation or cellular junction.

### KLK6 regulates neuronal synapse-related Appl2, Nav2, and Nrn1

**Fig. 6:**
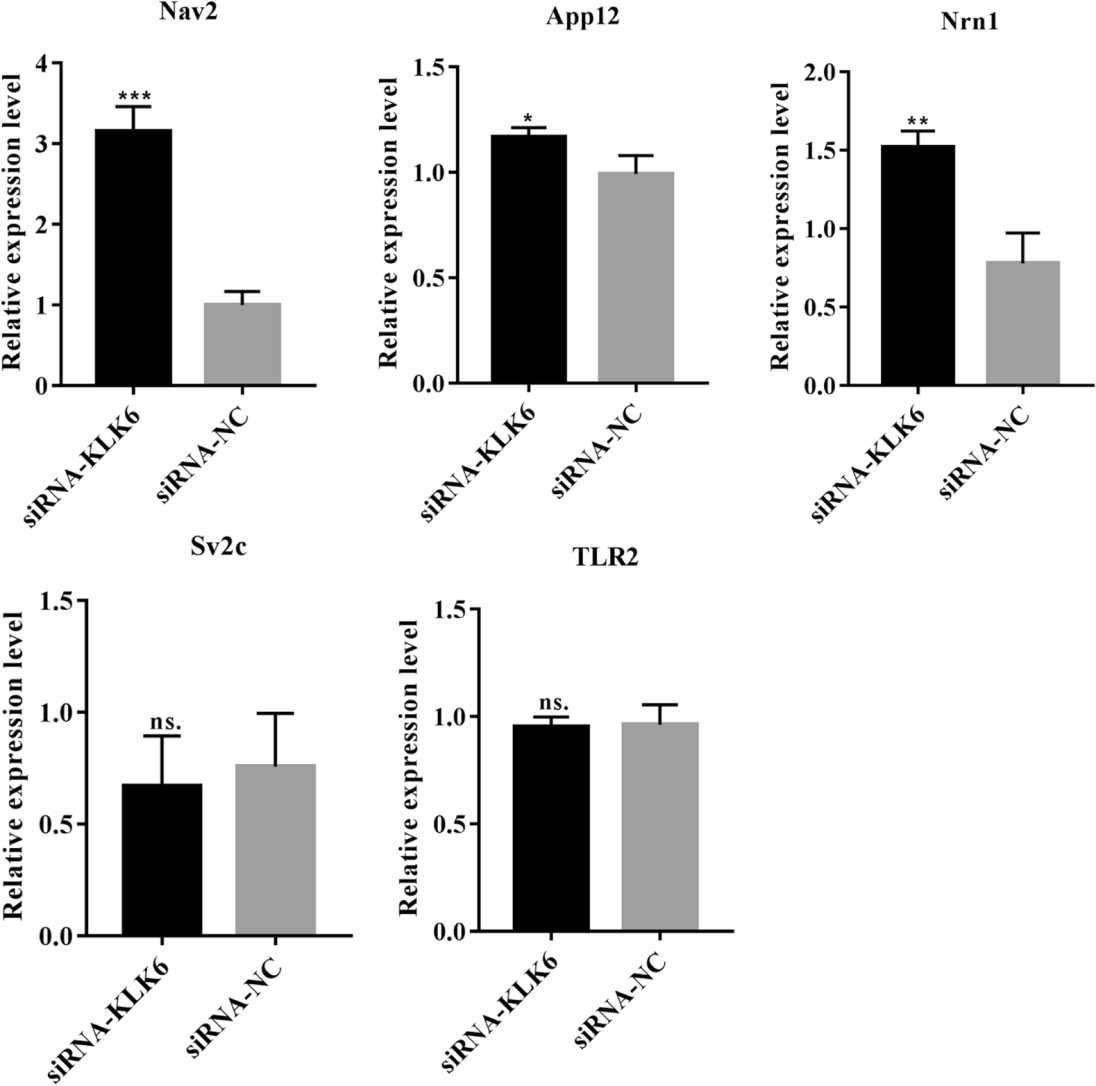
Verification of the expression of five DEGs using qRT-PCR. Appl2: adaptor protein, phosphotyrosine interaction, PH domain and leucine zipper containing 2. Nav2: neuron navigator 2. Nrn1: neuritin 1. TLR2: toll-like receptor 2. Sv2c: synaptic vesicle glycoprotein 2c. *indicates P < 0.05, **indicates P < 0.01, ***indicates P < 0.001, ns. indicates P > 0.05

Five DEGs related to immune regulation or cellular junction and positive response to siRNA-KLK6 were selected for qRT-PCR verification. The qRT-PCR results showed that adaptor protein, phosphotyrosine interaction, pH domain, leucine zipper containing 2 (Appl2), neuron navigator 2 (Nav2), and neuritin 1 (Nrn1) expression levels were significantly upregulated in the siRNA-KLK6 group, compared with those in the siRNA-NC groups (Fig. 6). The differences in the expression levels of toll-like receptor 2 and synaptic vesicle glycoprotein 2c were not significant between two groups (Fig. 6). Therefore, siRNA interference of KLK6 expression may affect the expressions of Appl2, Nav2, and Nrn1 to protect against damage to the synapse structure.

## Discussion

Hydrocephalus is a neurological disease in which CSF abnormally accumulates in the ventricles and causes abnormal expansion of the ventricles. CSF produced by the intraventricular choroid plexus flows through the aqueduct to the fourth ventricle. It then flows to the subarachnoid space, where the free communication between the ventricle and the subarachnoid space is expressed as CH [22]. In the United States alone, untreated hydrocephalus has a mortality rate of > 50%, with an estimated annual treatment cost in children of $2 billion [23]. However, some traditional therapies for CH have saved the lives of a limited number of patients, and the clinical treatment of CH slowly progresses due to the unclear molecular mechanism of CH. Herein, following initial screening by quantitative proteomics we obtained KLK6, and neuronal cell experiments further confirmed that regulation of Appl2, Nav2, and Nrn1 expression by KLK6 is the potential molecular mechanism of CH.

In this study, significantly differentially expressed KLK6 was screened based on quantitative proteomics, and KLK6 was found to co-localize with neurons in vivo, inhibiting neuronal cell activity, differentiation, and synaptic growth. KLK6 is a member of the KLK-related peptidase family, whose members are prominent in various neurological disorders. For example, KLK8 inhibition increases the number of neuronal progenitor cells and neuroplasticity-promoting interaction partners and promotes SH-SY5Y cell proliferation [24]. KLK7 deficiency aggravates the pathological features of amyloid-β deposited by astrocytes in AD [25]. Both KLK6 and KLK10 expression were elevated in CSF of patients with AD and associated with CSF-Tau, as shown by fluoro-d-glucose-positron emission tomography [26]. Shaw and Diamandis revealed that the distribution and expression of 15 human kallikrein members were significantly different in human tissue, among which KLK6 was dominant in both the brain and CNS [27]. Based on the research of KLK family members and our experimental results, that is, KLK6 expression is upregulated in the CH group, we believe that KLK6 participates in the progression of CH. Moreover, the present results on KLK6 are consistent with those of previous studies. Scarisbrick et al. corroborated that KLK6 enzyme cascade mediates spinal cord injury, including inhibition of neurite extension and cell adhesion [28]. Diamandis et al. found that the KLK6 level in CSF of patients with AD was approximately threefold higher than that in controls [29]. The findings of these studies support our conclusions and confirm that KLK6 is involved in neurological disorders.

In the present study, we found that inhibiting the expression of KLK6 led to the upregulation of Appl2, Nav2, and Nrn1 expression in neurons, which may be the potential mechanism of KLK6 inhibition to protect neuronal synaptic structures from damage. Appls are multifunctional adaptor proteins; Appl2 is an isoform of Appl1 that mediates the PI3K/Akt cascade to promote cortical neuronal survival [30] and also contributes to the modulation of synaptic plasticity via coupling neuronal activity with chromatin remodeling [31]. Thus, it is reasonable that Appl2 is involved in siRNA-KLK6-mediated synaptic structural recovery. Nav2 mRNA localizes to synaptic junctions and functions in neurite outgrowth, axonal elongation, and migration of the external granular layer neurons, and Nav2 deficiency can lead to abnormal cerebellar development [32]. Thus, Nav2 may be involved in siRNA-KLK6-mediated synaptic structural restoration. Moreover, a previous study reported that Nrn1 was involved in neurite outgrowth, which could reverse synaptic defects and cognitive function impairment in mice [33]. Nrn1 overexpression attenuated apoptosis, promoted axonal regeneration of retinal ganglion cells, and improved optic nerve crush rats [34]. Thus, upregulation of Nrn1 expression mediated by siRNA-KLK6 was conducive to synaptic structural remodeling. Consequently, interference of KLK6 expression improves the number of neurons and synapses in patients with CH, and the mechanism may be achieved by regulating Appl2, Nav2, and Nrn1 expression.

In conclusion, proteomic analysis on CSF found that KLK6 expression was significantly upregulated in CSF of patients with CH compared with patients without CH. KLK6 was confirmed to be highly expressed in the neuronal cells of CH rats, and KLK6 was shown to inhibit synaptic protein expression and synapse formation. Moreover, after the interference of KLK6 expression, the transcriptome profile was obtained, which showed that KLK6 may affect the neuronal cell structure by regulating the expression of Appl2, Nav2, and Nrn1. Our data might provide a new target and theoretical basis for the treatment of CH after SAH.

## Supplementary Information

**Additional file 1: Figure S1.** SDS-PAGE was performed to separate proteins extracted from CSF of the patients. Lanes 2, 7, and 8 represented patients with CH. Lanes 1, 3, 4, 5, and 6 represented patients without CH.

**Additional file 2: Figure S2.** Confidence of MS2 data. Distribution of peptides (A), peptide length (B), molecular weight of proteins (C), and protein sequence coverage (D).

**Additional file 3: Table S1.** Clinical profiles of patients with SAH.

**Additional file 4: Table S2.** Comparison between two groups in regards to comorbidities.

**Additional file 5: Table S3.** Details of the primers used in this study.

**Additional file 6: Table S4.** Statistical results of protein identification.

**Additional file 7: Table S5.** Detailed information for DEPs.

**Additional file 8: Table** **S6.** GO biological process and KEGG enrichment for significant DEPs.

**Additional file 9: Table S7.** Transcriptome sequencing quality control.

## Data Availability

The RNA-seq data generated in this study is available in the Sequence Read Archive (SRA) under accession numbers PRJNA719985 on the NCBI. The spectral data were deposited to the Figshare website (https://figshare.com/account/login) with the dataset https://doi.org/10.6084/m9.figshare.12335288. Other data that supports the findings of this study are available from the corresponding author upon reasonable request.

## Abbreviations

CH: Communicating hydrocephalus
CSF: Cerebrospinal fluid
ACSF: Artificial CSF
SAH: Subarachnoid hemorrhage
KLK: Kallikrein 6
DEPs: Differentially expressed proteins
CNS: Central nervous system
AD: Alzheimer’s disease
LC–MS/MS: Liquid chromatography–tandem mass spectrometry
FDR: False discovery rate
GO: Gene ontology
KEGG: Kyoto Encyclopedia of Genes and Genomes
PCA: Principal component analysis
NeuN: Neuronal nuclei
MAP-2: Microtubule-associated protein 2
DEG: Differentially expressed genes
FC: Fold-change
Appl2: Adaptor protein, phosphotyrosine interaction, PH domain, leucine zipper containing 2
Nav2: Neuron navigator 2
Nrn1: Neuritin 1
