# Supplementary figures and images for: Proteomics and functional study reveal kallikrein-6 enhances communicating hydrocephalus
Source: Clin Proteomics. 2021 Dec 16;18:30. doi: 10.1186/s12014-021-09335-9 (PMC8903716; doi:10.1186/s12014-021-09335-9)

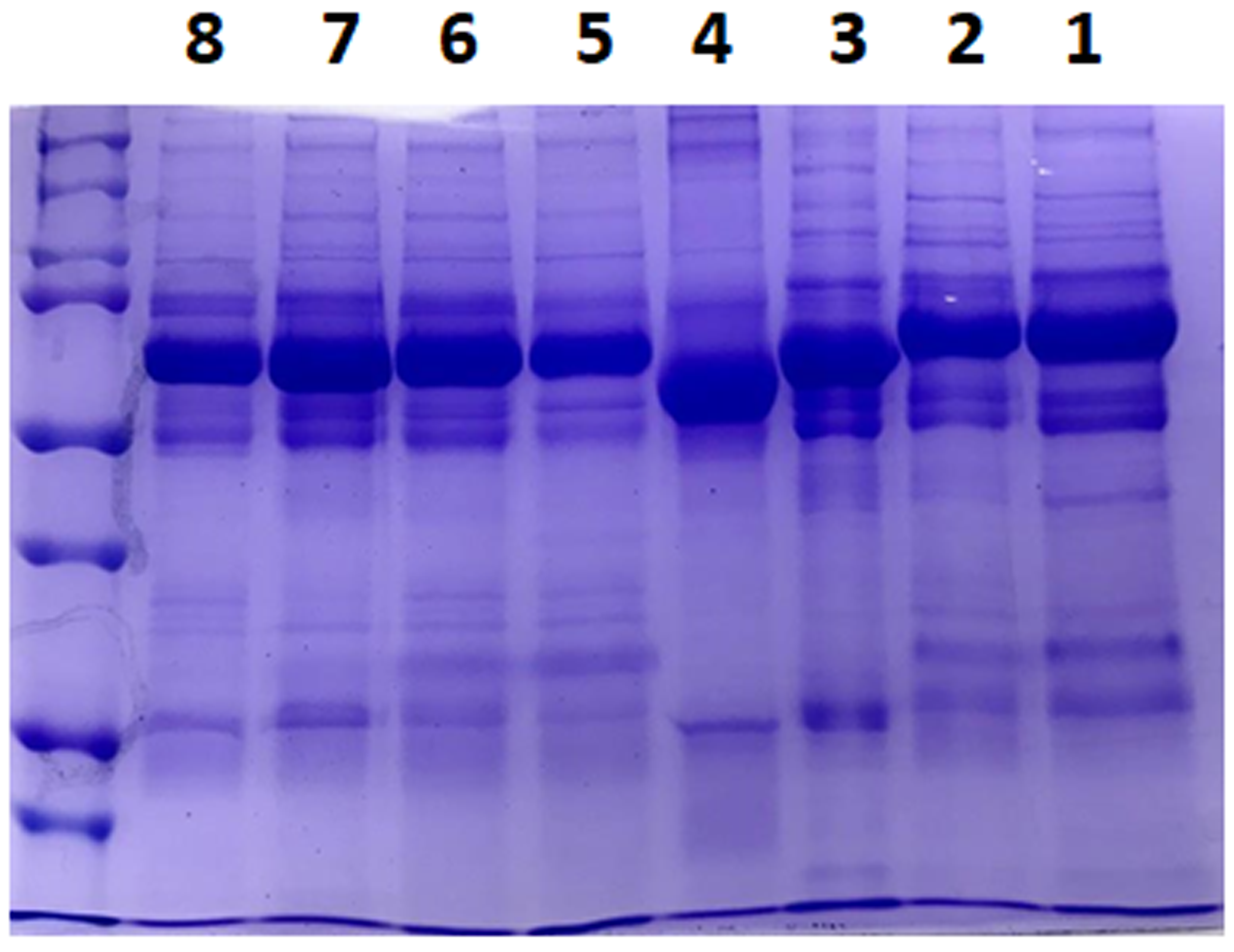

Supplement: Supplementary file 1 — Additional file 1: Figure S1. SDS-PAGE was performed to separate proteins extracted from CSF of the patients. Lanes 2, 7, and 8 represented patients with CH. Lanes 1, 3, 4, 5, and 6 represented patients without CH. [file 12014_2021_9335_MOESM1_ESM.tif]

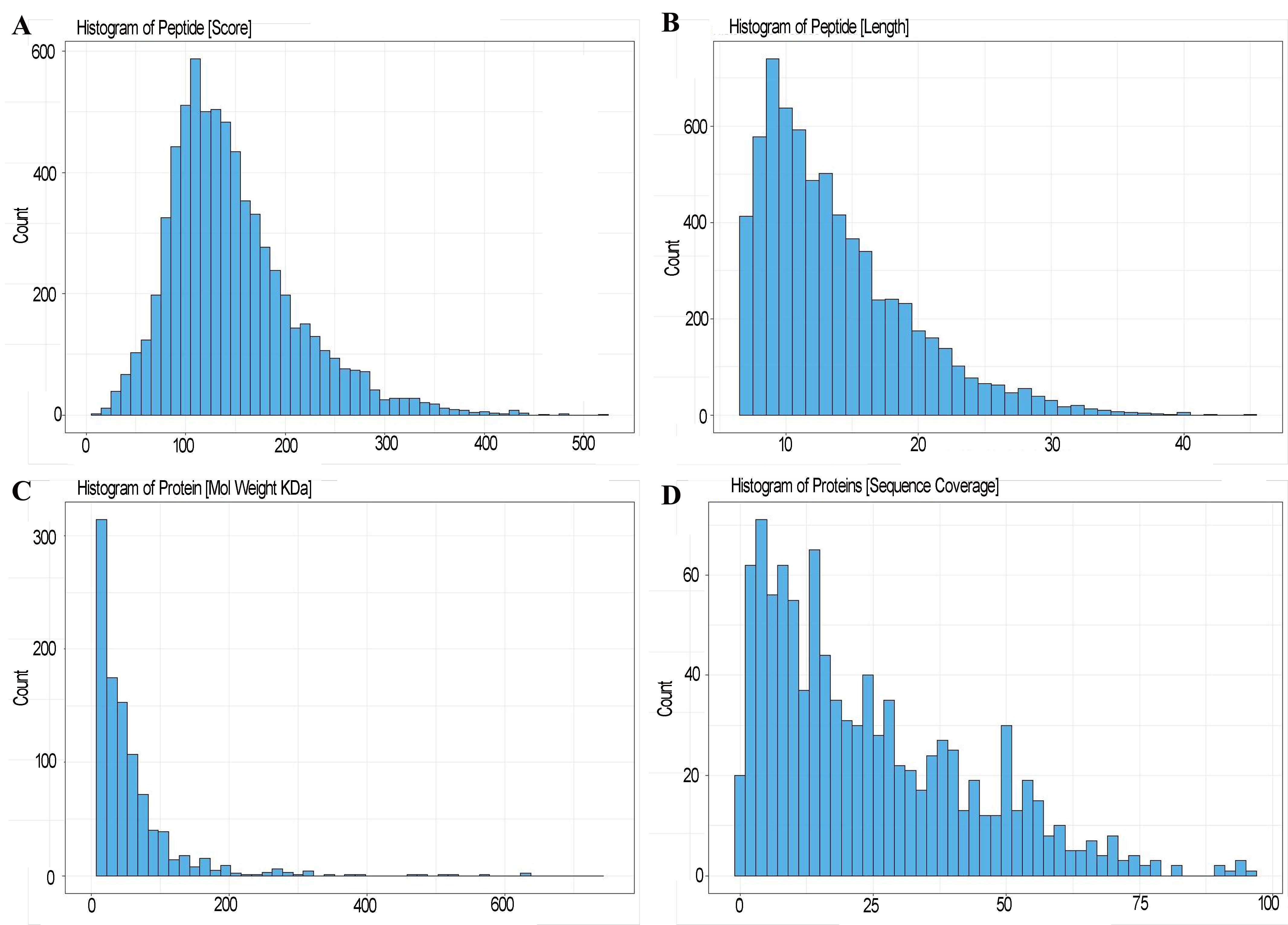

Supplement: Supplementary file 2 — Additional file 2: Figure S2. Confidence of MS2 data. Distribution of peptides (A), peptide length (B), molecular weight of proteins (C), and protein sequence coverage (D). [file 12014_2021_9335_MOESM2_ESM.tif]
