# Supplementary material for: Proteomics and functional study reveal kallikrein-6 enhances communicating hydrocephalus
Source: Clin Proteomics. 2021 Dec 16;18:30. doi: 10.1186/s12014-021-09335-9 (PMC8903716; doi:10.1186/s12014-021-09335-9)
Supplement: Supplementary file 3 — Additional file 3: Table S1. Clinical profiles of patients with SAH. [file 12014_2021_9335_MOESM3_ESM.docx]

**Additional file 3: Table S1.** Clinical profiles of patients with SAH.

| No. | Age | Sex | Aetiology | Fisher | WFNS | | Time of shunt (days) | |
| --- | --- | --- | --- | --- | --- | --- | --- | --- |
| 1 | 74 | M | ICA aneurysm | 4 | | IV | | 30 |
| 2 | 26 | F | PICA aneurysm | 4 | | V | | 30 |
| 3 | 68 | M | Basil aneurysm | 4 | | III | | 31 |
| 4 | 66 | M | PICA aneurysm | 4 | | III | | 35 |
| 5 | 67 | M | ICA aneurysm | 4 | | III | | 28 |
| 6 | 54 | F | C6A aneurysm | 4 | | III | | 45 |
| 7 | 52 | F | PICA aneurysm | 3 | | III | | 55 |
| 8 | 69 | F | MA aneurysm | 4 | | III | | 38 |
| 9 | 58 | M | C6A aneurysm | 4 | | IV | | 29 |
| 10 | 59 | M | PICA aneurysm | 4 | | V | | 18 |
| 11 | 25 | M | ACA aneurysm | 4 | | IV | | 15 |
| 12 | 41 | M | ACA aneurysm | 3 | | I | | n/a |
| 13 | 34 | M | C6A aneurysm | 4 | | III | | NA |
| 14 | 47 | M | PICA aneurysm | 4 | | V | | NA |
| 15 | 35 | M | ACA aneurysm | 2 | | II | | NA |
| 16 | 59 | F | PICA aneurysm | 3 | | III | | NA |
| 17 | 63 | F | PICA aneurysm | 4 | | IV | | NA |
| 18 | 27 | F | ICA aneurysm | 2 | | I | | NA |
| 19 | 38 | F | PCA aneurysm | 4 | | IV | | NA |
| 20 | 61 | M | PICA aneurysm | ND | | ND | | NA |

The sample of shaded in red is used for proteomic sequencing, sample 1, 2, and 3 were served as an analyzed group, sample 13, 14, 15, 16, and 17 were served as control group. SAH: Subarachnoid hemorrhage, ACA: anterior cerebral artery, ICA: internal carotid artery, PCA: posterior cerebral artery, PICA: posterior inferior cerebellar artery, ND: not determined, n/a: not available, NA: not applicable, WFNS: World Federation of Neurological Surgeons grade of subarachnoid hemorrhage.
