# Supplementary material for: Proteomics and functional study reveal kallikrein-6 enhances communicating hydrocephalus
Source: Clin Proteomics. 2021 Dec 16;18:30. doi: 10.1186/s12014-021-09335-9 (PMC8903716; doi:10.1186/s12014-021-09335-9)
Supplement: Supplementary file 4 — Additional file 4: Table S2. Comparison between two groups in regards to comorbidities. [file 12014_2021_9335_MOESM4_ESM.docx]

**Additional file 4: Table S2.** Comparison between two groups in regards to comorbidities

| **Group** | **CH** | **Control** | **P value** |
| --- | --- | --- | --- |
| Subjects for MS | n = 3 | n = 3 | – |
| Subjects for ELISA | n = 9 | n = 3 | – |
| Sex (female/male) | 4/8 | 4/4 | 0.18 |
| Age (years) (mean, range) | 54.9 (25–74) | 46.1 (27-66) | 0.23 |
| ***Comorbidities*** | Total (n = 12) | Total (n = 6) | – |
| Communicating hydrocephalus | n = 12, 100% | n = 0, 0% | 2.21E-05 |
| Hypertension | n = 7, 58% | n = 3, 50% | 0.74 |
| History of smoking | n = 2, 17% | n = 2, 33.3% | 0.42 |
| Pneumonia | n = 5, 41.7% | n = 2, 33.3% | 0.73 |
| Early Cerebral Ischemia | n = 2, 17% | n = 0, 0% | 0.29 |
| Diabetes mellitus | n = 3, 25% | n = 3, 50% | 0.29 |
| Hypercholesterolemia | n = 6, 8.3% | n = 4, 66.7% | 0.50 |

Statistical method for age was t test, for sex (female) and comorbidities were *X^2^*, P < 0.05 indicated statistical significance.
