# Supplementary material for: Proteomics and functional study reveal kallikrein-6 enhances communicating hydrocephalus
Source: Clin Proteomics. 2021 Dec 16;18:30. doi: 10.1186/s12014-021-09335-9 (PMC8903716; doi:10.1186/s12014-021-09335-9)
Supplement: Supplementary file 5 — Additional file 5: Table S3. Details of the primers used in this study. [file 12014_2021_9335_MOESM5_ESM.docx]

**Additional file 5: Table** **S3.** Details of the primers used in this study.

| Name | Sequences (5’ – 3’) |
| --- | --- |
| GAPDH-F | TGGAGAAACCTGCCAAGTATGAT |
| GAPDH-R | TCAAAGGTGGAAGAATGGGAGT |
| KLK6-F | CCTTTCCAAGCTGCCCTCTA |
| KLK6-R | GGAAAGTCTCAGTTTGCCGT |
| App12-F | TGGCAAAATACAGCAGGCTC |
| App12-R | GCGTTCAGTGCACAGTAGTA |
| Nav2-F | TGCGCCAGTATCTGTCCAA |
| Nav2-R | GCAGTTAAGCAGTCCGTTGA |
| Nrn1-F | CACAGCTCTTACGGATTGCC |
| Nrn1-R | TGCCGCAGAGTTCGAATAAG |
| TLR2-F | TCTGCTCCTGTGAACTCCTG |
| TLR2-R | AGGATCAACAGGAGAAGGGC |
| Sv2c-F | GGGCTTTGGCTTCTTGAACG |
| Sv2c-R | AACCTGGGTTCTTGTGTCGG |
| siRNA NC | UUCUCCGAACGUGUCACGUTTACGUGACACGUUCGGAGAATT |
| siRNA-KLK6-249 | GGUCGGAAGAUCAGGAUAATTUUAUCCUGAUCUUCCGACCTT |
| siRNA-KLK6-472 | GGACAGGACUAUUGUCCAUTTAUGGACAAUAGUCCUGUCCTT |
| siRNA-KLK6-249 | GCACUCAUAUCAGAUGGAUTTAUCCAUCUGAUAUGAGUGCTT |
