# Supplementary material for: Proteomics and functional study reveal kallikrein-6 enhances communicating hydrocephalus
Source: Clin Proteomics. 2021 Dec 16;18:30. doi: 10.1186/s12014-021-09335-9 (PMC8903716; doi:10.1186/s12014-021-09335-9)
Supplement: Supplementary file 6 — Additional file 6: Table S4. Statistical results of protein identification. [file 12014_2021_9335_MOESM6_ESM.docx]

**Additional file 6: Table S4.** Statistical results of protein identification.

| Total spectra | Spectra | Peptides | Protein groups |
| --- | --- | --- | --- |
| 388562 | 41139 | 6815 | 1008 |
