# Supplementary material for: Proteomics and functional study reveal kallikrein-6 enhances communicating hydrocephalus
Source: Clin Proteomics. 2021 Dec 16;18:30. doi: 10.1186/s12014-021-09335-9 (PMC8903716; doi:10.1186/s12014-021-09335-9)
Supplement: Supplementary file 7 — Additional file 7: Table S5. Detailed information for DEPs. [file 12014_2021_9335_MOESM7_ESM.docx]

| Gene name | Protein name | Majority protein ID | P value | Fold Change (FC) | Groups |
| --- | --- | --- | --- | --- | --- |
| IGHG1 |  | A0A087WV47 | 0.007 | 0.80 | p<0.05,0.67<FC<1.5 |
| CD44 | CD44 antigen | H0YD13 | 0.026 | 0.36 | p<0.05,FC<0.67 |
| TIMP1 | Metalloproteinase inhibitor 1 | Q5H9A7 | 0.039 | 2.25 | p<0.05,FC>1.5 |
|  | Ig heavy chain V-III region TIL/ TUR/ WAS/ POM | P01765 | 0.043 | 0.63 | p<0.05,FC<0.67 |
| TF | Serotransferrin | P02787 | 0.021 | 0.69 | p<0.05,0.67<FC<1.5 |
| HRG | Histidine-rich glycoprotein | P04196 | 0.049 | 0.52 | p<0.05,FC<0.67 |
|  | Ig kappa chain V-III region CLL | P04207 | 0.031 | 0.60 | p<0.05,FC<0.67 |
| SERPIND1 | Heparin cofactor 2 | P05546 | 0.022 | 0.74 | p<0.05,0.67<FC<1.5 |
| APOA4 | Apolipoprotein A-IV | P06727 | 0.016 | 0.49 | p<0.05,FC<0.67 |
| SERPINA4 | Kallistatin | P29622 | 0.048 | 0.54 | p<0.05,FC<0.67 |
| KLK6 | Kallikrein-6 | Q92876 | 0.043 | 0.65 | p<0.05,FC<0.67 |

**Additional file 7:** **Table S5. Detailed information for DEPs**
