# Supplementary material for: Proteomics and functional study reveal kallikrein-6 enhances communicating hydrocephalus
Source: Clin Proteomics. 2021 Dec 16;18:30. doi: 10.1186/s12014-021-09335-9 (PMC8903716; doi:10.1186/s12014-021-09335-9)
Supplement: Supplementary file 8 — Additional file 8: Table S6. GO biological process and KEGG enrichment for significant DEPs. [file 12014_2021_9335_MOESM8_ESM.docx]

**Additional file 8: Table S6.** GO biological process and KEGG enrichment for significantly DEPs

| Term ID | Term description | Observed gene name | Fold Enrichment | Adjust P value |
| --- | --- | --- | --- | --- |
| GO:0002576 | platelet degranulation | TF, SERPINA4, HRG, TIMP1 | 72.45738943 | 4.911 |
| GO:0010951 | negative regulation of endopeptidase activity | SERPINA4, HRG, SERPIND1, TIMP1 | 61.67860422 | 4.700998 |
| GO:0051216 | cartilage development | CD44, TIMP1 | 63.24670433 | 1.556407 |
| GO:0022617 | extracellular matrix disassembly | CD44, TIMP1 | 49.0994152 | 1.447982 |
| GO:0006935 | chemotaxis | HRG, SERPIND1 | 30.58652095 | 1.246588 |
| hsa04066 | HIF-1 signaling pathway | TF, TIMP1 | 28.6625 | 1.262203 |

TF: serotransferrin, SERPINA4: kallistatin, HRG: histidine-rich glycoprotein, TIMP1: metalloproteinase inhibitor 1, SERPIND1: heparin cofactor 2, CD44: CD44 antigen.
