# Supplementary material for: Proteomics and functional study reveal kallikrein-6 enhances communicating hydrocephalus
Source: Clin Proteomics. 2021 Dec 16;18:30. doi: 10.1186/s12014-021-09335-9 (PMC8903716; doi:10.1186/s12014-021-09335-9)
Supplement: Supplementary file 9 — Additional file 9: Table S7. Transcriptome sequencing quality control. [file 12014_2021_9335_MOESM9_ESM.docx]

**Additional file 9: Table S7.** Transcriptome sequencing quality control.

| Sample Name | Total Reads  After | Total Base  After | Reads Filter % | Base Filter % | GC % | Mapped Reads^a^ | Mapped Rate |
| --- | --- | --- | --- | --- | --- | --- | --- |
| siKLK6 1 | 82176374 | 12315597840 | 0.962 | 0.961 | 50.5 | 73861763 | 0.899 |
| siKLK6 2 | 111527448 | 16715774871 | 0.963 | 0.963 | 50 | 101501886 | 0.91 |
| siKLK6 3 | 74958180 | 11234803153 | 0.961 | 0.960 | 50 | 69084070 | 0.922 |
| siNC 1 | 74058474 | 11098933622 | 0.956 | 0.955 | 49.5 | 68664635 | 0.927 |
| siNC 2 | 78809034 | 11811574248 | 0.958 | 0.957 | 49.5 | 73598508 | 0.934 |
| siNC 3 | 65104784 | 9755346824 | 0.954 | 0.953 | 50 | 60030867 | 0.922 |

^a^ Clean reads mapped to reference genome of rnor6.
